# Diagnostic Utility of Paton’s Folds and Quantitative OCT Metrics in Pediatric Papilledema

**DOI:** 10.1016/j.ophtha.2026.01.002

**Published:** 2026-01-12

**Authors:** Jonathan Zhou, Raj Vadhul, David Rogers, Hersh Varma

**Affiliations:** 1 The Ohio State University College of Medicine, Columbus, Ohio; 2 Department of Ophthalmology, The Ohio State University, Columbus, Ohio; 3 Department of Ophthalmology, Nationwide Children’s Hospital, Columbus, Ohio

**Keywords:** Papilledema, Pseudopapilledema, OCT, peripapillary wrinkling, retinal folds

## Abstract

**Purpose:**

We evaluated the diagnostic accuracy of peripapillary retinal folds (RFs) and peripapillary wrinkling (PPW) in distinguishing papilledema (PE) from pseudopapilledema (PPE) in pediatric patients using spectral-domain OCT (SD-OCT). We also identified diagnostic threshold values for retinal nerve fiber layer (RNFL) thickness and optic nerve head volume (ONHV).

**Design:**

Single-center, hospital and clinic-based, retrospective, cross-sectional study.

**Participants:**

We included 84 eyes from 42 pediatric patients with PE and 92 eyes from 46 pediatric patients with PPE.

**Methods:**

Two masked ophthalmologists (H.V. and D.R.) independently reviewed transverse and en face SD-OCT images to assess for RF and PPW. We manually segmented retinal layers and measured RNFL thickness and ONHV. We performed receiver operating curve (ROC) analysis with area under the curve (AUC) to determine optimal diagnostic thresholds. We assessed inter-rater reliability using Cohen’s kappa.

**Main Outcome Measures:**

Sensitivity and specificity for RF and PPW; optimal diagnostic thresholds for RNFL and ONHV in distinguishing PE from PPE in pediatric patients.

**Results:**

We observed RFs or PPW in 90.2% of PE eyes and 38.6% of PPE eyes (*P* < 0.0001). Retinal folds demonstrated higher sensitivity (79.2%) but lower specificity (67.4%), whereas PPW showed higher specificity (92.9%) and lower sensitivity (43.5%) in distinguishing PE from PPE in pediatric patients. Transverse OCT had the highest probability of detecting RFs as a marker for PE, whereas en face OCT was more reliable at detecting PPW. Receiver operating characteristic analysis identified optimal PE screening thresholds of ≥163 μm for RNFL (AUC = 0.908) with 78% sensitivity and 93% specificity and a threshold of ≥5.43 mm^3^ for ONHV (AUC = 0.855) with 73% sensitivity and 90% specificity. Inter-rater agreement was higher for RF on transverse OCT (κ = 0.61) and higher for PPW on en face OCT (κ = 0.54).

**Conclusions:**

Peripapillary wrinkling is specific to PE in pediatric patients and an excellent rule-in tool. Transverse SD-OCT reliably detects Paton’s folds, particularly RF. However, the limited specificity of RF and higher inter-rater variability of rare, subtle findings underscore the importance of incorporating quantitative OCT metrics. Retinal nerve fiber layer thickness and ONHV offer strong discriminative power and objective thresholds to support clinical decision-making. In borderline cases, clinicians can improve diagnostic confidence by integrating fold detection with RNFL and ONHV analysis, additional ancillary testing, and clinical history with exam to create a more accurate, noninvasive approach to distinguish PE from PPE in children.

**Financial Disclosure(s):**

Proprietary or commercial disclosure may be found after the references.

Papilledema (PE) is a neuro-ophthalmic emergency characterized by optic disc swelling secondary to elevated intracranial pressure (ICP). It may signal a serious underlying condition such as idiopathic intracranial hypertension (IIH), intracranial mass, hydrocephalus, or meningitis. Among these, IIH is the most common cause with an estimated incidence of 0.32 to 0.63 per 100 000 children.^1^ Despite increasing referrals for suspected PE based on either symptoms or fundus examination, the actual prevalence of true PE among referrals is low, with one study citing an incidence of just 2 patients in 34 referrals.^2^ Timely recognition of PE is crucial, because untreated intracranial hypertension can cause significant morbidity from vision loss and chronic headaches. Conversely, unnecessary workup of pseudopapilledema (PPE) can lead to expensive and invasive diagnostic procedures that are distressing for patients and their families.^3^

Pseudopapilledema refers to optic disc elevation without elevated ICP, and it is typically associated with structural anomalies such as optic disc drusen, peripapillary hyperreflective ovoid mass-like structures (PHOMS), or crowded optic nerves. Optic disc drusen, by far the most common cause, are deposits within the optic nerve head (ONH) found in less than 1% of pediatric patients.^4^ In younger children, these drusen tend to be deeply buried and less calcified,^5^ further complicating the diagnostic challenge. Clinical examination alone is frequently insufficient, and various adjunctive tools have been used to improve diagnostic accuracy, including visual field testing,^6^ fundus autofluorescence,^7^ fluorescence angiography,^8^ and B-scan ultrasonography.^9,10^ One promising avenue of investigation is the use of OCT imaging to identify secondary features of elevated ICP: peripapillary retinal folds (RFs) and peripapillary wrinkling (PPW), which were initially described clinically as Paton’s folds. Peripapillary wrinkling is undulating distortions of the inner retinal surface, visible as irregularities along the internal limiting membrane (ILM) contour in the peripapillary region. Retinal folds are structural, undulating deformations of the inner or outer retina that may occur anywhere between the ILM and Bruch’s membrane and are more widely spaced than PPW. Both PPW and RF are hypothesized to be caused by mechanical stress, most commonly from ONH swelling such as in PE.^11^ However, RFs have been observed in nonarteritic anterior ischemic optic neuropathy^12^ and after retinal detachment surgery.^13^

Spectral-domain OCT (SD-OCT) is a rapid, noninvasive, radiation-free, and widely available imaging modality that allows detailed visualization of the ONH and surrounding retina. Previous studies, including those by Sibony et al,^14,15^ have demonstrated that OCT patterns of Paton’s folds correlate with elevated ICP. Nevertheless, the specificity of these findings to true PE, particularly in the pediatric population, is less clearly defined.^16^

The primary purpose of our study is to quantify the prevalence of Paton’s folds in pediatric patients with PE and PPE using SD-OCT and to compare the diagnostic utility of transverse (cross-sectional) and en face OCT imaging in identifying these features. We also offer numerical cutoffs and area under the curve (AUC) for average retinal nerve fiber layer (RNFL) thickness and optic nerve head volume (ONHV) as diagnostic adjuncts. Our goal is to expand the noninvasive diagnostic toolkit for clinicians assessing optic disc edema in children.

## Methods

### Study Design and Ethical Approval

This was a single-center, retrospective, cross-sectional chart-review study conducted at a tertiary pediatric institution. The study received approval from the Institutional Review Board at Nationwide Children’s Hospital. The requirement for informed consent was waived by the Institutional Review Board because this retrospective study involved the analysis of existing clinical data and posed minimal risk to participants. All procedures adhered to the Declaration of Helsinki and complied with the Health Insurance Portability and Accountability Act.

### Eligibility Criteria

Eligible participants were pediatric patients (<18 years) who underwent SD-OCT with enhanced-depth imaging using a Heidelberg Spectralis HRA+OCT machine for evaluation of suspected optic disc elevation. Clinical and imaging data were obtained from routine care between November 2023 and February 2025. Patients were identified using EPIC SlicerDicer queries that included the following diagnostic codes: PE, benign intracranial hypertension, PPE, optic disc drusen, and congenitally crowded optic nerves. All included patients were initially referred to a pediatric IIH clinic or evaluated in the emergency department for suspected PE. Patients were excluded if their OCT imaging was performed more than 7 days after initiating acetazolamide or more than 24 hours after cerebrospinal fluid diversion surgery (e.g., ventriculoperitoneal shunt) or if OCT image quality was inadequate.

### Participants

Two diagnostic cohorts were defined: One cohort consisted of patients with a clinical diagnosis of PE confirmed by elevated lumbar puncture opening pressure and clinical improvement after treatment with reduction in both RNFL thickness and optic disc edema at follow-up. The second cohort consisted of patients with optic disc elevation due to structural anomalies (e.g., PHOMS, buried optic disc drusen, congenitally crowded discs). These patients showed clinical stability and minimal variation in average RNFL thickness on serial OCT exams during follow-up and were diagnosed with PPE based on characteristic imaging and examination findings.

### Data Extraction and Management

Careful resegmentation of the ILM and Bruch’s membrane layers were performed for all 73 slices of each ONH scan before grading for each eye. Optic nerve head volume was derived using a 3-mm radius that was manually centered over the optic nerve. Average RNFL thicknesses were obtained after manual resegmentation of peripapillary retinal scans.

**Figure 1.**
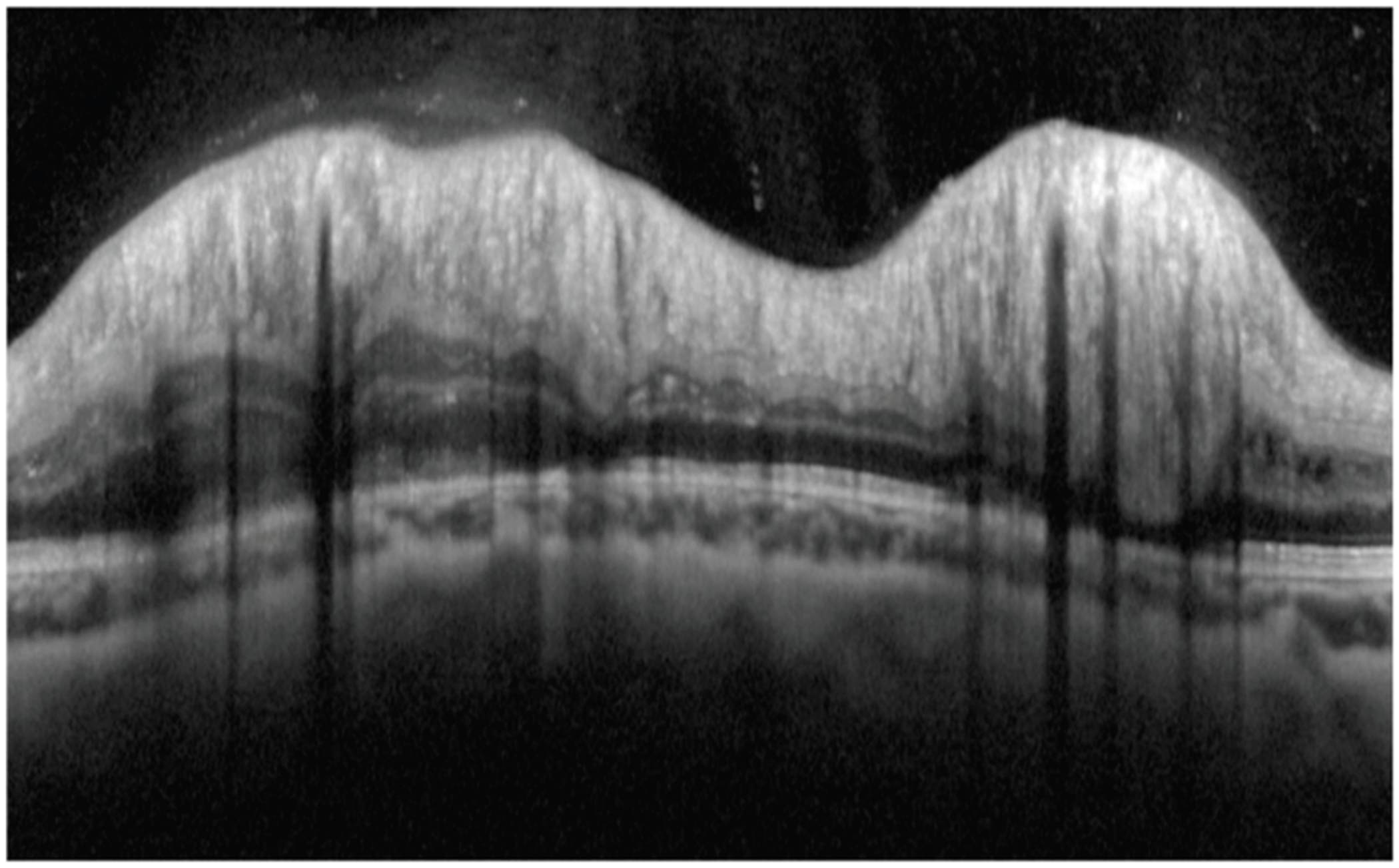
Retinal folds (RFs) on a 360-degree peripapillary retina OCT scan.

**Figure 2.**
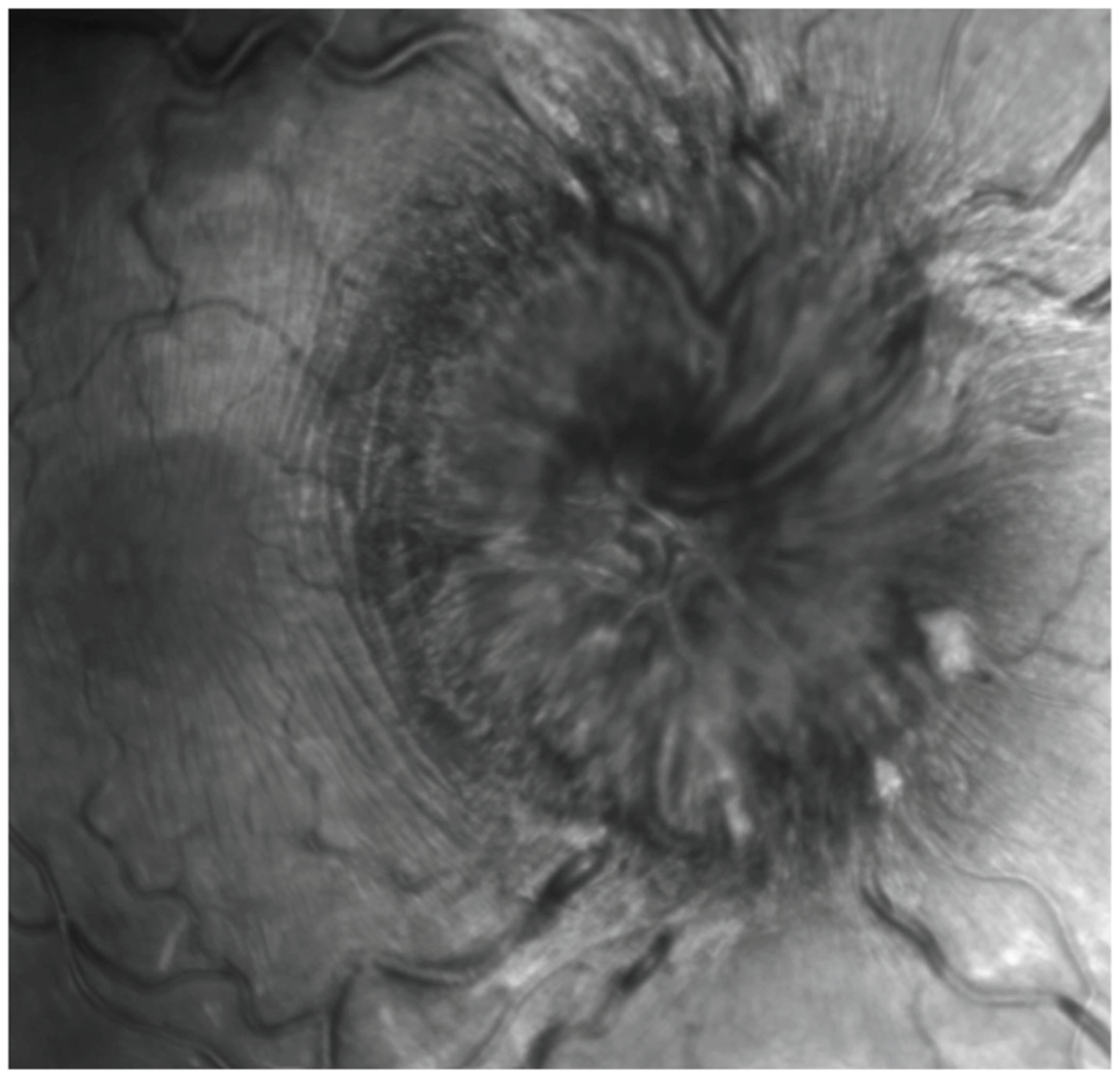
Peripapillary wrinkling (PPW), seen as concentric lines surrounding the optic nerve head (ONH).

Two masked ophthalmologists (D.R. and H.V.) independently graded each patient’s OCT scans for the presence or absence of PPW and RF on both en face and transverse views in each eye. Graders agreed on grading criteria and used a standardized protocol for reviewing scans to identify PPW and RF. The protocol included first examining the transverse OCT view for folds and then examining the en face view. If a fold was noted on either view, its location was corroborated on the other view. An RF (Fig 1) was defined as a sequence of undulations within the retinal layers, rather than a single peak and trough, particularly excluding single peaks that were immediately adjacent to structures such as optic nerve drusen. A PPW (Fig 2) was defined as a series of fine, repetitive undulations confined to the inner-most retinal layers (ILMs) within the peripapillary region, not due to adjacent structures such as PHOMS. Reviewers were blinded to patient diagnosis and history during image assessment. Diagnostic test characteristics (sensitivity, specificity) were calculated for both PPW and RF, combining grades from both graders. Descriptive statistics were stored and calculated using Excel for participant demographics (Table 1). Sensitivities and specificities by image category for each individual grader are shown in Tables 2 and 3.

### Data Synthesis and Analysis

The primary outcome was the prevalence of PPW and RFs in each diagnostic group at the time of OCT scans. We then compared the sensitivity and specificity of PPW and RF for PE between en face and transverse scans to determine which image categories had the highest reliability. The following method was used to account for the loss of independence when using both eyes per patient and 2 graders per scan: To analyze the data, we fit a binomial Generalized Linear Mixed model with a logit link function. The model was used to predict the presence of identified items (the response variable) based on the fixed effect of image category (RF or PPW seen on en face or transverse views). To account for individual differences and the nonindependence of paired eyes, we included a random intercept for each participant in whom the eye was nested. The model provides odds ratios (ORs) for each image category. To measure the agreement between the 2 reviewers on the presence or absence of an indicator, we used Cohen’s Kappa (κ) to establish a single, overall measure of inter-reviewer agreement (rating of an image). The Kappa statistic was applied to obtain a single snapshot of reviewer consistency, and then the Generalized Linear Mixed model was used to appropriately analyze the complex, nested structure of the data. Additionally, we produced receiver operating characteristic (ROC) curves for average RNFL thickness and ONHV at a 3-mm radius, with optimal threshold values calculated using Youden’s Index. The ROCit package in R was used to create ROC curves and identify Youden indices (J) and the corresponding optimal threshold values for RNFL thickness and ONH volume.^17^

## Results

A total of 84 eyes from 42 pediatric patients with PE and 92 eyes from 46 pediatric patients with PPE were included (Table 1). Eyes were excluded if scan quality was poor or if patients with PE did not have an elevated lumbar puncture opening pressure. The median age was 13.4 years (range, 3–17) for the PE group and 12.0 years (range, 5–15) for the PPE group. The sex ratio was skewed toward female patients in the PE group (female:male = 2:1), and the PPE group had a 1:1 sex ratio. Forty patients with PPE had optic nerve drusen, 8 patients had PHOMS, 4 patients had anomalous appearing nerves, and 1 patient had megalopapillae. Six of 8 patients with PHOMS had concomitant optic nerve drusen.

The mean RNFL thickness was significantly greater in PE eyes (right eye: 247 ± 62.9 μm; left eye: 239 ± 60.2 μm) compared with PPE eyes (right eye: 122 ± 30.5 μm; left eye: 124 ± 34.0 μm; *P* < 0.001). The median Frisen grade was 2+ in the right eye and 3+ in the left eye in the PE group, and 1+ bilaterally in the PPE group. Of note, in the absence of an established PPE grading scale, we used the Frisen scale. The average ONHV in PE was 6.22 mm^3^ versus 3.96 mm^3^ in PPE. Median RNFL thickness was 247 μm in PE versus 117 μm in PPE. Linear regression analysis showed that an increase of approximately 24 cmH_2_O correlated to an increase of approximately +1 Frisen score, although the R^2^ value of this association was low at 0.23. Additional subgroup analysis by age (e.g., 3–10 years vs 11–17 years) did not reveal a significant difference in RNFL thickness or ONHV between patients within each diagnostic cohort.

Tables 2 and 3 are contingency tables organized by fold (RF or PPW), grader, and image category (transverse or en face OCT). Tables 2 and 3 reflect the frequency of structural OCT folds observed across all eyes, with each eye independently graded by 2 blinded reviewers (i.e., 42 patients, 84 eyes, yielding 376 assessments). Paton’s folds (PPW or RF) were observed in 90.2% of PE eyes versus 38.6% of PPE eyes (*P* < 0.0001). Retinal folds were also found in 79.2% of PE eyes and 32.6% of PPE eyes (*P* < 0.0001), whereas PPW was present in 43.5% and 7.1% of PE and PPE eyes, respectively (*P* < 0.0001). The sensitivity and specificity of RF for PE were 79.2% and 67.4%, respectively; for PPW, they were 43.5% and 92.9%, respectively. The presence of concurrent PPW and RF had a sensitivity of 34.9% and a specificity of 96.7% at detecting PE.

When comparing en face with transverse views, Paton’s folds were detected in 59.5% of en face OCT images of PE eyes versus 24.5% of PPE eyes (*P* < 0.0001). Paton’s folds were detected in 90.0% of transverse OCT images of PE eyes versus 32.1% of PPE eyes (*P* < 0.0001). The specificity of Paton’s folds on en face OCT for PE and PPE was 75.5% and 67.9%, respectively.

Our Generalized Linear Mixed model indicated that RFs found on transverse scans were the most reliable and predictive finding of PE. By comparison, the odds of identifying RF on en face views were 93% lower (OR, 0.07), whereas the odds of identifying PPW on en face or transverse views were 85% lower (OR, 0.15) and 90% lower (OR, 0.10), respectively.

Cohen’s unweighted kappa, a measure of inter-rater agreement, was substantial (κ = 0.61) for RF on transverse OCT, moderate for PPW on transverse (κ = 0.54), moderate (κ = 0.54) for PPW on en face, and fair (κ = 0.18) for RF on en face OCT for patients with PE. In the PPE cohort, agreement was substantial (κ = 0.62) for RF on transverse OCT, moderate (κ = 0.52) for RF on en face OCT, moderate (0.43) for PPW on en face OCT, and fair (κ = 0.30) for PPW on transverse OCT.

**Figure 3.**
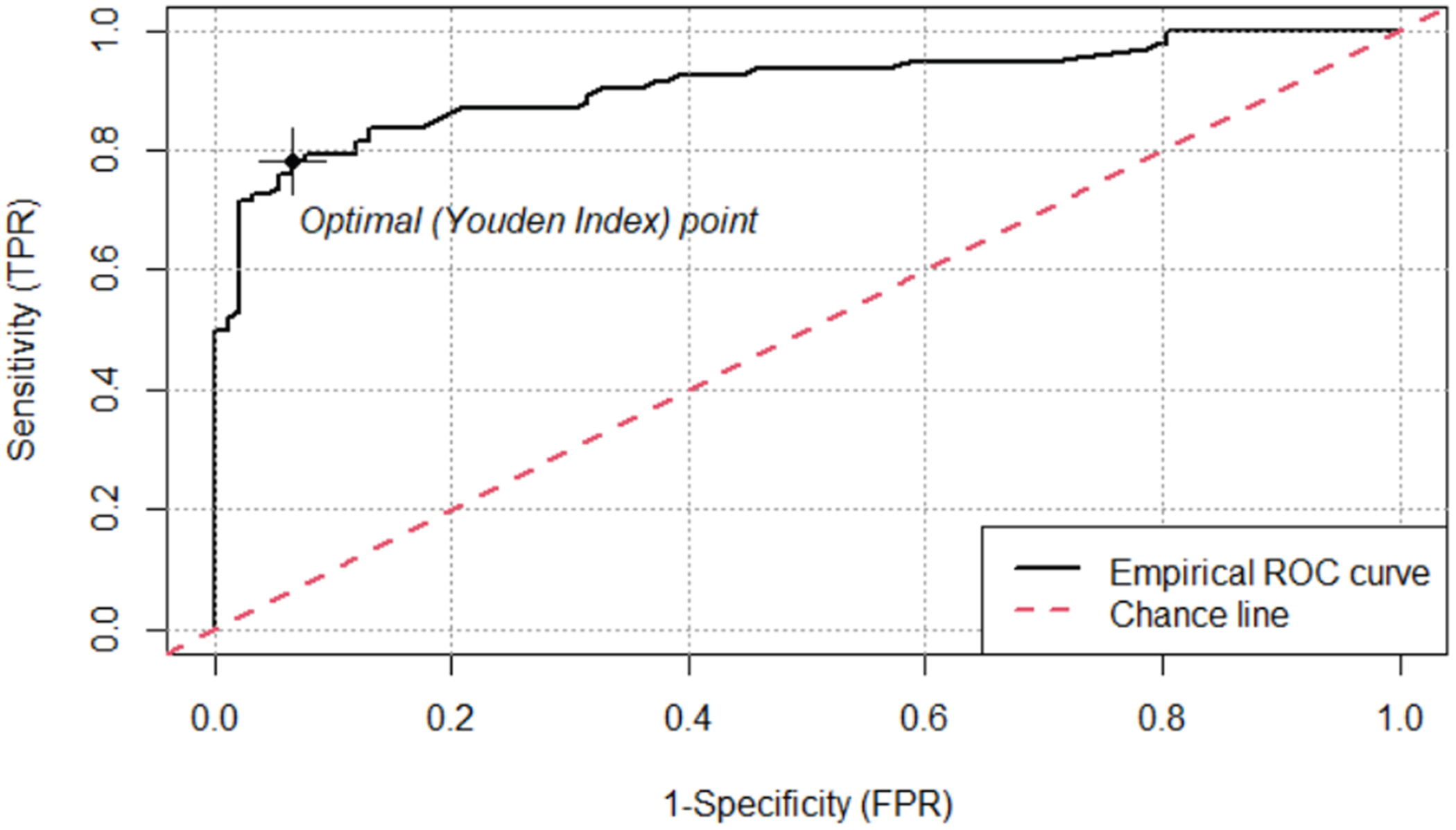
Receiver operating characteristic (ROC) curve for average retinal nerve fiber layer (RNFL) thickness. The ROC curve for average RNFL thickness has an area under the curve (AUC) of 0.908. A threshold of 163 μm as determined by Youden’s Index, optimizes sensitivity at 78% and specificity at 93%. No patients with pseudopapilledema (PPE) were observed with a threshold >255 μm, and no patients with papilledema (PE) were observed with a threshold <101 μm. FPR = false-positive rate; TPR = true positive rate.

**Figure 4.**
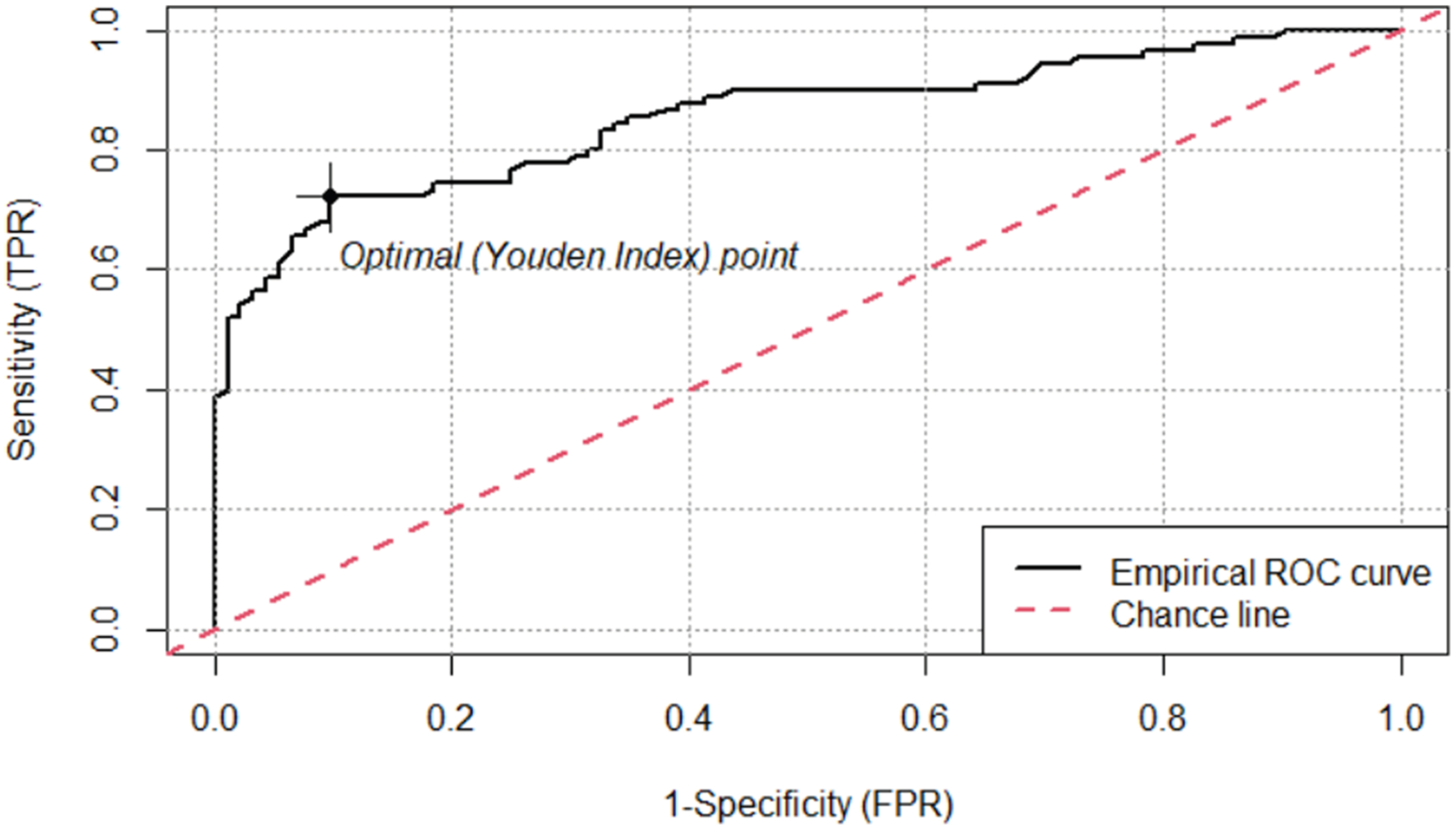
The receiver operating characteristic (ROC) curve for optic nerve head (ONH) volume. An ROC curve of optic nerve head volume (ONHV) has an area under the curve (AUC) of 0.855. A threshold of 5.43 mm^3^ optimizes sensitivity at 73% and specificity at 90%. No patients with pseudopapilledema (PPE) were observed with a threshold >6.74 mm^3^, and no patients with PE were observed with a threshold <3.17 mm^3^. FPR = false-positive rate; TPR = true positive rate.

The ROC analysis showed that RNFL had an area under the curve (AUC) of 0.908, and a threshold of 163 μm optimized sensitivity at 78% and specificity at 93% (Fig 3). Optic nerve head volume had an AUC of 0.855, and a threshold of 5.43 mm^3^ optimized sensitivity at 73% and specificity at 90% (Fig 4).

## Discussion

There is a common precept that Paton’s folds generally do not occur in many etiologies of PPE such as optic disc drusen^14^ and that their presence is therefore a reliable diagnostic marker for PE. However, few studies have even attempted to quantify their prevalence in PPE,^11^ even fewer in pediatrics, making their true diagnostic utility difficult to evaluate. Our study challenges this precept by demonstrating that the diagnostic utility of Paton’s folds varies dramatically based on the specific fold type (PPW vs. RF) and orientation of the scan axis (transverse vs. en face). Here, we describe the specific contexts in which Paton’s folds, as detected by SD-OCT, can provide important diagnostic clues in differentiating PE from PPE in children.

Peripapillary wrinkling, seen best on en face OCT, showed high specificity (92.9%) for PE, but its low sensitivity (43.5%) limits its utility beyond a rule-in tool for PE. In contrast, RFs, best seen on transverse views, were the most often visible feature of PE, offering higher sensitivity (79.2%) but reduced specificity (67.9%). Retinal folds are thus reasonable to consider as a moderately sensitive screening tool on OCT. It is important to recognize that RFs are structural undulations that likely reflect nonspecific patterns of biomechanical stress and are thus less definitive indicators of elevated ICP.^12,13^

When comparing imaging modalities, transverse OCT outperformed en face OCT in detecting Paton’s folds, showing higher sensitivity (78.4% vs 58.5%) with a mild tradeoff in specificity (67.9% vs. 75.5%). The improved visibility of retinal layers on cross-sectional imaging as opposed to en face views may explain its superior diagnostic performance.^14^ Should RFs be used as a screening tool, it may be helpful to optimize their sensitivity. For example, imaging the eye in adduction may increase fold detection.^14^ Transverse SD-OCT scans must be oriented perpendicular to folds for their full height and profile to be reliably appreciated.

Given the limitations of using Paton’s folds in isolation as diagnostic markers, it is important to integrate quantitative OCT metrics such as RNFL thickness and ONHV as complementary tools in clinical evaluation. In our analysis, RNFL thickness and ONHV demonstrated strong discriminative ability, with AUC values of 0.908 and 0.855, respectively. The optimal threshold for average RNFL thickness identified in this study was 163 μm, which is higher than a 125 μm cutoff previously reported in the literature.^18^ This threshold optimizes sensitivity at 78% and specificity at 93%. The pediatric ONHV threshold identified in this study of 5.43 mm^3^ also exceeds an adult value of 3.97 mm^3^ as a discriminatory cutoff in the literature.^19^ This threshold optimizes sensitivity at 73% and specificity at 90%.

These elevated thresholds, along with the relatively high prevalence of Paton’s folds, increased RNFL thickness, and increased ONHV in PPE cases, suggest that distinguishing true PE from PPE is more challenging in pediatric populations than in adults, underscoring the benefit of a multimodal diagnostic approach. Clinicians should synthesize multiple OCT features, combining structural fold detection with RNFL and ONHV measurements to improve diagnostic confidence. Our findings support expanding the upper limit of “normal” RNFL thickness into the 150 to 160 μm range in pediatric patients. In the absence of a suspicious clinical context, values below this threshold may support a decision to defer further invasive workup for elevated ICP. In cases where RNFL or ONHV values are borderline or inconclusive, careful inspection of both transverse and en face SD-OCT images for Paton’s folds may help refine the pretest probability of PE and guide management decisions. Ultimately, because no single imaging modality can yet definitively distinguish PE from PPE, we encourage clinicians to interpret OCT findings in the context of a full history and exam, additional ancillary testing, and, in some cases, longitudinal follow-up or systemic investigation. Taken together, this study provides objective benchmarks to add to the noninvasive framework for ancillary testing with SD-OCT to distinguish PE from PPE in pediatric patients.

### Limitations

This study also has several limitations. First, its retrospective design introduces the possibility of selection bias and limits control over imaging acquisition. Clinical documentation may have varied between providers. Second, although performed by 2 independent reviewers using a standardized protocol, the grading of RFs and PPW is inherently subjective. Furthermore, although graders were blinded to diagnosis and the presence of structures such as PHOMS, drusen, or vasculature does not exclude the diagnosis of PE, visualization of these features may have introduced bias into grading for the presence of folds. Although there was substantial inter-rater agreement in the PE cohort for the presence of RF on transverse OCT (κ = 0.61), there was poor agreement (κ = 0.18) for RF on en face OCT and moderate agreement for PPW seen on either en face (κ = 0.54) or transverse (κ = 0.54) views. In the PPE cohort, agreement was substantial (κ = 0.62) for RF on transverse OCT, moderate (κ = 0.52) for RF on en face OCT, moderate (κ = 0.43) for PPW on en face OCT, and fair (κ = 0.30) for PPW on transverse OCT. Taken together, these kappa values suggest that although the observed structural features are all useful, consistent identification of them is difficult when such features are rare and subtle, which subjects their grading to influence by image quality, acquisition, or interpretation. We recommend reliably identifying RF on transverse views and scanning for PPW on en face views.

This study was conducted at a single tertiary pediatric center, which may limit generalizability to other clinical settings or to adult populations. The study focused on PPW and RFs rather than choroidal folds. Additionally, patients with PE were occasionally imaged up to 7 days after initiation of treatment with acetazolamide or 1 day after the placement of a lumboperitoneal shunt, which may have affected the prevalence or visibility of structural features. Finally, although SD-OCT can provide high-resolution, enhanced-depth imaging, it is possible that subtle or deep structural changes may have gone undetected without other modalities such as B-scan ultrasonography or fluorescein angiography.

## Conclusions

Our findings reinforce the utility of SD-OCT in the diagnosis of PE and provide numerical context for interpretation of structural OCT findings. Additionally, we offer diagnostic thresholds for 2 quantitative, reliable metrics in tracking PE, average RNFL thickness, and ONHV. Ultimately, a multivariate approach that combines structural folds with RNFL thickness, ONHV, other ancillary testing metrics, clinical history, and a full exam will offer improved diagnostic confidence when evaluating optic disc elevation in children.

## Figures and Tables

**Table 1.** Baseline Characteristics and OCT Metrics by Diagnosis

	Papilledema	Pseudopapilledema	*P* Value
**Age at scan, yrs**	12.4 ± 3.6 (3—17)	10.4 ± 2.7 (5—15)	<0.0041
**Sex**	Female: 28, Male: 14	Female: 23, Male: 23	<0.1136
**Median average RNFL thickness**	247 (102—435 μm)	117 (79—251 μm)	<0.0001
**Median ONHV (3-mm radius)**	6.22 (3.21—9.65 μm)	3.96 (2.72—6.64 μm)	<0.0001
**Lumbar puncture opening pressure or average duration follow-up**	40.0 ± 10.2	1 yr, 4 mos (3 mos to 4 yrs)	

Summary statistics for age, sex, RNFL thickness, and lumbar puncture data by diagnosis group with ranges. *P* values were calculated by chi-square tests or Welch’s *t* tests of unequal variance. ONHV = optic nerve head volume; RNFL = retinal nerve fiber layer.

**Table 2.** Frequency of Retinal Folds on Transverse Spectral-Domain OCT: Grader 1

	Retinal Folds Present	Retinal Folds Absent	Total
**PE**	71 (TP)	13 (FN)	84
**PPE**	31 (FP)	61 (TN)	92
**Total**	102	74	176
Frequency of Retinal Folds on Transverse Spectral-Domain OCT: Grader 2.			
	*Retinal Folds Present*	*Retinal Folds Absent*	*Total*

**PE**	62 (TP)	22 (FN)	84
**PPE**	29 (FP)	63 (TN)	92
**Total**	91	85	176

Diagnostic utility of retinal folds (RFs) seen on transverse spectral-domain OCT (SD-OCT) by grader 1. Sensitivity: 84.5%, specificity: 66.3%. Diagnostic utility of RFs seen on transverse SD-OCT by grader 2. Sensitivity: 73.8%, specificity: 68.5%. FN = false negative; FP = false positive; PE = papilledema; PPE = pseudopapilledema; TN = true negative; TP = true positive.

**Table 3.** Frequency of Peripapillary Wrinkles on En Face Spectral-Domain OCT: Grader 1

	PPW Present	PPW Absent	Total
**PE**	38 (TP)	46 (FN)	84
**PPE**	4 (FP)	88 (TN)	92
**Total**	42	134	176
Frequency of Peripapillary Wrinkles on En Face Spectral-Domain OCT: Grader 2.			
	*PPW Present*	*PPW Absent*	*Total*

**PE**	35 (TP)	49 (FN)	84
**PPE**	9 (FP)	83 (TN)	92
**Total**	44	132	176

Diagnostic utility of PPW seen on en face SD-OCT by grader 1. Sensitivity: 45.2%, specificity: 95.7%. Diagnostic utility of PPW seen on en face SD-OCT by grader 2. Sensitivity: 41.7%, specificity: 90.2%. FN = false negative; FP = false positive; PE = papilledema; PPE = pseudopapilledema; PPW = peripapillary wrinkling; TN = true negative; TP = true positive.

## Abbreviations and Acronyms:

AUC: area under the curve
ICP: intracranial pressure
IIH: idiopathic intracranial hypertension
ILM: internal limiting membrane
ONH: optic nerve head
ONHV: optic nerve head volume
OR: odds ratio
PE: papilledema
PHOMS: peripapillary hyperreflective ovoid mass-like structures
PPE: pseudopapilledema
PPW: peripapillary wrinkling
RF: retinal fold
RNFL: retinal nerve fiber layer
ROC: receiver operating curve
SD-OCT: spectral-domain OCT
